# The Targetable Epigenetic Tumor Protein EZH2 is Enriched in Intraocular Medulloepithelioma

**DOI:** 10.1167/iovs.16-20463

**Published:** 2016-11

**Authors:** Sarah E. Avedschmidt, Anna M. Stagner, Ralph C. Eagle, George J. Harocopos, Yali Dou, Rajesh C. Rao

**Affiliations:** 1 Department of Pathology, University of Michigan, Ann Arbor, Michigan, United States; 2 Department of Ophthalmology, Massachusetts Eye and Ear Infirmary, Harvard Medical School, Boston, Massachusetts, United States; 3 Department of Pathology, Massachusetts General Hospital, Harvard Medical School, Boston, Massachusetts, United States; 4 Department of Ophthalmic Pathology, Wills Eye Hospital, Thomas Jefferson University, Philadelphia, Pennsylvania, United States; 5 Department of Ophthalmology and Visual Sciences, Washington University School of Medicine, St. Louis, Missouri, United States; 6 Department of Pathology and Immunology, Washington University School of Medicine, St. Louis, Missouri, United States; 7 Department of Biological Chemistry, University of Michigan, Ann Arbor, Michigan, United States; 8 Comprehensive Cancer Center, University of Michigan, Ann Arbor, Michigan, United States; 9 Department of Ophthalmology and Visual Sciences, W. K. Kellogg Eye Center, University of Michigan, Ann Arbor, Michigan, United States; 10 A. Alfred Taubman Medical Research Institute, University of Michigan, Ann Arbor, Michigan, United States; 11 Section of Ophthalmology, Surgical Service, Veterans Administration Ann Arbor Healthcare System, Ann Arbor, Michigan, United States

**Keywords:** medulloepithelioma, epigenetics, EZH2

## Abstract

**Purpose:**

Intraocular medulloepithelioma (IM), the second most common primary neuroepithelial tumor of the eye, can lead to blindness in the affected eye and in rare cases, is deadly. Intraocular medulloepithelioma lacks targetable biomarkers for potential pharmacologic therapy. The purpose of this study was to identify actionable, tumor-specific proteins for potential diagnostic or therapeutic strategies. We hypothesize that the tumor-specific epigenetic enzyme EZH2 is selectively expressed in IM.

**Methods:**

We conducted a retrospective case series study of five IM from five eyes of four children and one adult. Hematoxylin and eosin (H&E) stains of sections from formalin-fixed, paraffin-embedded blocks of IM tumors were used to localize IM tumor cells in each case. Using an EZH2-specific antibody for immunohistochemistry, we semiquantitatively calculated the proportion of IM tumor cells positive for EZH2, and also assayed for EZH2 staining intensity.

**Results:**

We found that EZH2 was expressed in all IM cases but this protein was absent in nontumor ciliary body or retinal tissues. However, not all IM tumor cells expressed EZH2. Similar to retinoblastoma, moderately to poorly differentiated (primitive appearing) IM tumor cells strongly expressed EZH2; expression was weaker or absent in areas of well-formed neuroepithelial units.

**Conclusions:**

To our knowledge, this is the first study to identify an actionable tumor-specific maker, EZH2, in IM. Our findings point to the possibility of exploring the potential of EZH2 inhibitors, already in clinical trials for other cancers, for IM.

Intraocular medulloepithelioma (IM) is an uncommon primitive neoplasm that arises from the embryonic neuroepithelium that forms the ciliary body, iris, retina, or optic nerve head.^1^ Intraocular medulloepithelioma is the second most common primary intraocular cancer in childhood after retinoblastoma, with an incidence of one case per 450,000 to 1,000,000.^2^ More than 80% of cases are diagnosed before 7 years of age.^2^ Intraocular medulloepithelioma can lead to blinding complications, including cataract, retinal detachment, and neovascular glaucoma.^1^ No standard of care exists for IM; therapy can include brachytherapy, iridocyclectomy, or enucleation, depending on the size of the tumor, whether extraocular extension exists, and vision at the time of diagnosis.^1^ Unlike central nervous system (CNS) medulloepitheliomas, IMs rarely metastasize.^1,3^

Recently, new IM histopathologic biomarkers and genomic alterations have been reported. Such biomarkers include CRX and LIN28A, and recurrent mutations occur in *DICER1* and *KMT2D* genes.^2,4,5^ Nonetheless, the presence of these biomarkers or mutations does not point toward targeted therapies that could potentially reduce the morbidity associated with these tumors or treatments associated with them, such as blindness from glaucoma or enucleation. To address these unmet needs in IM as well as ocular/orbital/ocular adnexal cancers in general, we have applied genetic and epigenetic-based precision medicine strategies to retinoblastoma,^6^ orbital and ocular adnexal lymphoma,^7^ and cutaneous basal cell carcinoma.^8^

In those reports,^6–8^ we discovered that the epigenetic enzyme EZH2 is a key biomarker for aggressive forms of these cancers, including retinoblastoma, which, like IM, arises from neuroepithelial progenitor cells.^5,6^ EZH2, like KMT2D, is a chromatin modifier that methylates lysine residues on histone H3, which regulates gene expression, including tumor suppressors and oncogenes.^6^ Gain of function *EZH2* mutations and *EZH2* overexpression are frequent among cancers, and several small molecule EZH2 inhibitors have recently entered early phase clinical trials for nonophthalmic cancers such as lymphoma and advanced solid tumors.^2,7^ The goal of the current study is to explore whether the targetable epigenetic protein EZH2 is specifically enriched IM.

## Methods

This study was approved by the University of Michigan (Ann Arbor, MI, USA) institutional review board. Deidentified slides from IM cases were sent from Wills Eye Hospital (Thomas Jefferson University, Philadelphia, PA, USA) and Washington University in St. Louis (MO, USA). Slides recut from archived, paraffin-embedded, formalin-fixed blocks from one iridocyclectomy specimen and four enucleations for IM (four previously untreated, one with previous treatment) were analyzed. Each slide was stained with hematoxylin and eosin (H&E) and EZH2, as previously described,^6^ and EZH2 staining was qualitatively scored by two pathologists (SEA and AMS) on the basis of proportion of nuclei with positive staining (<30%, focal; 30%–70%, moderate; >70%, diffuse) and intensity (weak/moderate/strong).

## Results

**Figure 1:**
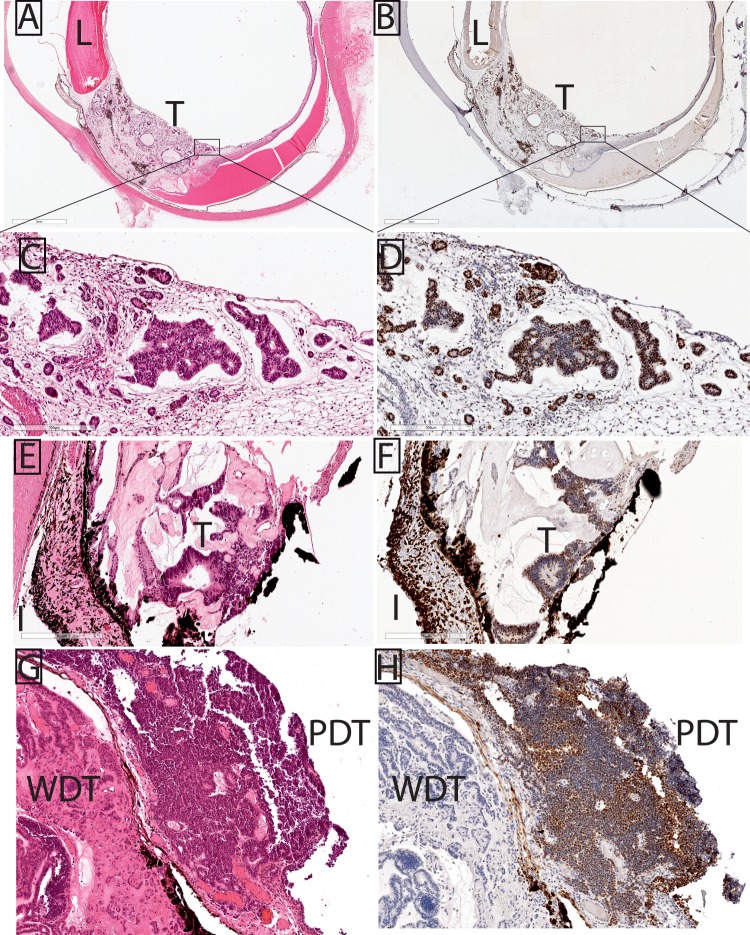
EZH2 immunolocalization to IM tumor cells in the anterior segment. Each H&E (**A**, **C**, **E**, **G**) section is matched with corresponding, EZH2-immunostained section (**B**, **D**, **F**, **H**). Insets in (**A**) and (**B**) correspond to high-power images in (**C**) and (**D**), respectively. (**A**, **C**) Enucleated globe contains a ciliary body tumor (T); behind the lens (L), a neoplastic membrane is also seen. (**B**, **D**) EZH2 localizes to IM tumor cells in the ciliary body. (**E**) In another enucleation specimen, neuroepithelial units extend posterior to the iris. (**F**) EZH2 is present in IM tumor (T) nuclei; the remainder of brown pigment represents melanosomes in epithelial and stromal cells of the iris (I). (**G**) An iridocyclectomy specimen contains both well-differentiated (WDT) and poorly differentiated, neuroblastic (PDT) regions of IM tumor. (**H**) The primitive, poorly differentiated portion of the tumor exhibits greater EZH2 staining than the well-differentiated region. *Scale bars*: (**A**, **B**) 3 mm, (**C**–**F**) 300 μm. Magnification in (**G**, **H**) is ×100.

**Figure 2:**
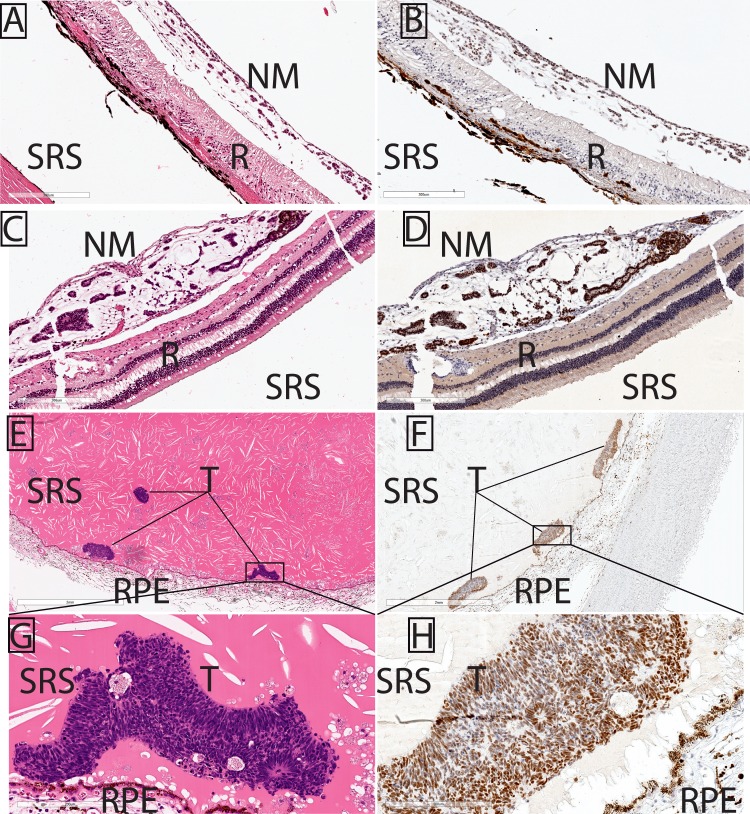
EZH2 immunolocalization to IM tumor cells in the posterior segment. Each H&E (**A**, **C**, **E**, **G**) section is matched with a corresponding, EZH2-immunostained section (**B**, **D**, **F**, **H**). (**A**–**D**) Intraocular medulloepithelioma tumor cells have formed neoplastic epiretinal membranes (NM). (**B**, **D**) EZH2 is present only in the neoplastic epiretinal membrane (NM) IM cell nuclei but is absent from nontumor retina (R). (**E**–**H**) Enucleated globe from a case with previous systemic chemotherapy and extraocular extension shows IM tumor (T) as multicellular IM neuroepithelial units in the subretinal space (SRS), anterior to the RPE. EZH2 localizes to IM tumor cells. *Scale bars*: (**A**–**D**) 300 μm, (**E**, **F**) 2 mm, (**G**, **H**) 200 μm.

This report focuses on the histopathologic findings relating to EZH2 immunohistochemistry in IM; limited clinical information (Table) was available for each case. All five IM tumors showed classic hallmarks of IM, including multilaminar rosettes or neurotubules, cords or festoons of cells, and small cellular units that were either solid or displayed different-sized lumens, surrounded by a loose mesenchymal tissue (Figs. 1, 2). EZH2 was present in each of the five tumors analyzed, and histopathologic findings relating to tumor grade, proportion of IM cells positive for EZH2 staining, and EZH2 staining intensity are summarized in the Table. EZH2 staining was restricted to IM cells, and was not present in any other cell type, including the nontumor ciliary body epithelial cells, neural retina, or retinal pigment epithelium (RPE; Figs. 1, 2). For instance, EZH2-positive IM tumor cells formed epiretinal membranes over EZH2-negative neural retina (Figs. 2A–D).

**Table:**
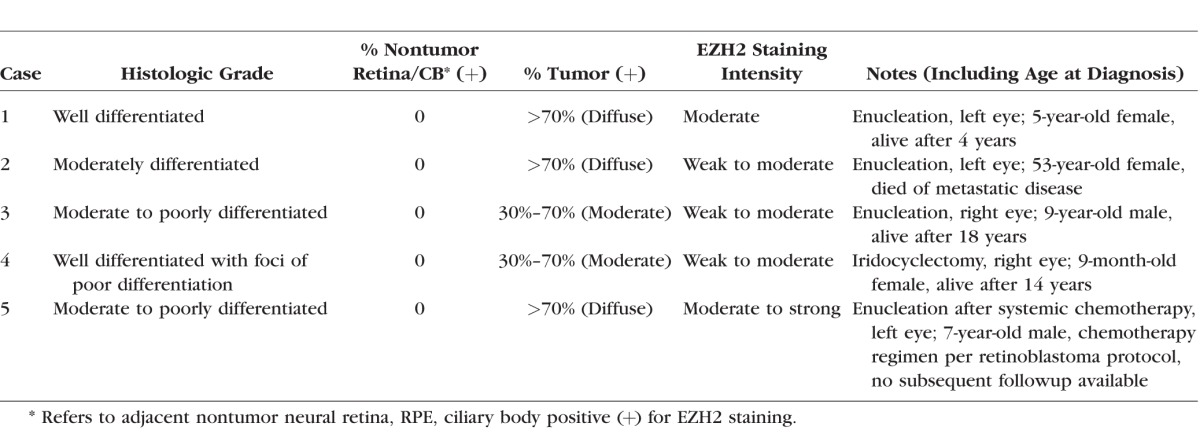
Summary of IM Cases

The proportion of IM cells staining positive for EZH2 was moderate to diffuse in each case, but occasionally varied within each tumor. EZH2 did not appear to mark all IM tumor cells. Similar to our previously reported findings in retinoblastoma, EZH2 appeared absent or only weakly positive in neuroepithelial units of the well-differentiated portions of the IM tumor, but was diffusely and strongly positive in moderately to poorly differentiated primitive/neuroblastic IM tumor cells (Figs. 1G, 1H). In the phthisical eye enucleated from a child with prior chemotherapy and orbital extension of IM, small foci of residual IM tumor cells are easily visible by EZH2 immunohistochemistry (Fig. 2E–H). Taken together, these data indicate that EZH2 is specifically enriched in IM, both in primary tumors and in a residual tumor after systemic chemotherapy. Importantly EZH2 was never found in surrounding nontumor tissues, such as neural retina, ciliary body, iris, and RPE cells.

## Discussion

To our knowledge, this is the first study to identify an actionable target in IM, the second most common primary intraocular cancer in children. This targetable epigenetic factor, EZH2, is a histone methyltransferase that catalyzes formation of H3K27me3, which is known to silence tumor suppressor gene expression, thereby promoting tumorigenesis.^6^

In our recent report of EZH2 expression in retinoblastoma (RB), we found that while EZH2 appears to be a specific marker for RB, it did not mark all RB cells. We noted that EZH2 protein is low or absent in focal regions of photoreceptor differentiation (e.g., fleurettes).^6^ Similarly, EZH2 does not mark all IM tumor cells, and appeared to have a predilection for moderately to poorly differentiated regions of the tumor. Why might this be? Our recent work linking EZH2 expression to normal fetal retinal development and retinoblastoma indicates that this epigenetic marker does not distinguish among fetal neuroretinal progenitor cells, retinoblastoma, and IM.^4^ Inasmuch as EZH2 is tightly linked to cellular proliferation,^8^ this epigenetic enzyme may simply be enriched in retinal cells undergoing rapid proliferation, which occurs during retinal development and tumorigenesis.

Despite the sample size of five cases, our study is one of the largest immunohistochemical series of IM to date, owing to the fact that IM is quite rare.^2–5^ Our findings have two major implications. One, EZH2 may serve as a diagnostic biomarker to detect invasive IM tumor cells in the ciliary body, retina, iris, and possibly optic nerve or extraocular tissues, similar to retinoblastoma. Second, because EZH2 is specifically expressed in IM tumor cells, but not other nontumor cells, EZH2 represents an attractive avenue for targeted therapy for intraocular cancers. Indeed, we found that small molecule EZH2 inhibitors related to those currently in early phase clinical trials, uniquely target retinoblastoma tumor cells but spare nontumor RPE cells in vitro. EZH2 inhibitors might have a similar effect on IM cells; however, no in vitro or in vivo models for IM exist. Future derivation of IM cell lines from enucleated eyes or establishment of animal models with conditional intraocular deletions of *DICER1* or *KMT2D* could enable a preclinical platform to test the efficacy of EZH2 inhibitors and other targeted strategies.^2,6^

The study of epigenetics in ocular and orbital diseases is in its infancy. The recent discovery of recurrent mutations in another histone methyltransferase, *KMT2D*, in IM highlights the importance of epigenetics in the tumorigenesis of this uncommon tumor.^2^ From studies of cancers in other parts of the body, therapies that target tumor addiction to epigenetic dysregulation, such as recurrent EZH2 overexpression or gain of function mutations, have emerged as one of the most promising strategies against cancer. For instance, in the last 2 years, several clinical studies (in the public domain, https://clinicaltrials.gov/ct2/results?term=ezh2) evaluating the use of EZH2 as a biomarker or therapeutic target in lymphoma and a variety of solid cancers have emerged. Our seminal studies detailing EZH2 dysregulation in RB, orbital and ocular adnexal lymphoma, and cutaneous basal cell carcinoma have uncovered “the tip of the iceberg” of the potential of using epigenetics to better understand how ocular, ocular adnexal, and orbital tumors form, and to develop novel therapeutics.^6–8^ Taken together, this study indicates EZH2 is a biomarker for IM, and highlights the possibility that EZH2 could be exploited as a therapeutic target for this pediatric tumor, a cancer that lacks biologically targeted treatments.
